# In what context and by which mechanisms can creative arts interventions improve wellbeing in older people? A realist review protocol

**DOI:** 10.3310/nihropenres.13746.1

**Published:** 2025-03-04

**Authors:** Alexandra Caulfield, Anne Ferrey, Nia Roberts, Jeremy Leslie-Spinks, Helle Mölsted Alvesson, Geoff Wong, Trish Greenhalgh

**Affiliations:** 1 University of Oxford Nuffield Department of Primary Care Health Sciences, Oxford, England, UK; 2 University of Oxford Bodleian Libraries, Oxford, England, UK; 3 Karolinska Institute Department of Global Public Health, Stockholm, Stockholm County, Sweden

**Keywords:** Creative arts, older people, wellbeing, mental health, creative health, creative ageing

## Abstract

**Background:**

In recent years, there has been growing interest at national and international policy level in the potential of creative arts to support individual and community wellbeing. Creative arts encompass a wide range of activities, including performing arts, visual arts, design and craft, literature, culture and digital and electronic arts. Participation in creative arts has been linked to lower mental distress, increased social connection, improved quality of life, personal growth and empowerment. Despite this, it remains unclear exactly how participation in creative arts interventions can improve wellbeing in older individuals. This realist review aims to synthesize evidence on how elements of creative arts interventions improve wellbeing amongst older people, in particular when, how, for whom and to what extent they work.

**Methods and analysis:**

This review will follow the RAMESES (Realist And Meta-narrative Evidence Syntheses: Evolving Standards) quality standards and Pawson’s five iterative stages to locate existing theories, search for evidence, select literature, extract data, and draw conclusions. It will be guided by stakeholder engagement with policymakers, practitioners, commissioners, and people with lived experience. A realist approach will be used to analyse data and develop causal explanations, in the form of context-mechanism-outcome-configurations (CMOCs), which explain how creative arts interventions impact wellbeing in older people. The CMOCs will be organised into one or more programme theories. Our refined programme theory will then be used to develop guidance for service providers of creative arts who want to use their services to improve wellbeing of older people.

**Ethics and dissemination:**

This research will comply with the UK Policy Framework for Health and Social Care Research. Dissemination will be guided by our stakeholder group, building on links with policymakers, commissioners, providers, and the public. A final stakeholder event focused on knowledge mobilisation will aid development of recommendations.

PROSPERO registration CRD42024580770.

## Plain Language Summary

Creative activities (such as singing, painting, crafts, writing etc.) can help people feel better and improve their wellbeing. Taking part in these activities may make it easier to meet others, create a sense of belonging, a feeling of achievement and improve quality of life. In recent years, the World Health Organisation and the UK government have encouraged research to explore these benefits. They want to understand how creative activities can improve health for people and their communities. At the moment, we don’t know much about exactly how these creative activities might affect wellbeing. This review aims to identify how creative activities work to improve wellbeing in older adults, and in what kind of circumstances they work best. We hope to use our results to develop practical guidance for people who teach or provide creative arts activities. This will help improve the wellbeing of older people living in the community.

## Introduction

### Creative arts and health

In recent years, there has been growing interest in the potential of creative arts to support individual and community wellbeing. Creative arts encompass a wide range of activities, including performing arts, visual arts, design and craft, literature, culture and digital and electronic arts ^1^. Involvement may be receptive (e.g. watching a film, visiting a museum) or participatory (e.g. painting, writing), depending on the degree to which the participant is involved in the creative process, though in reality engagement likely falls across a spectrum between the two. Across the UK, 9.4 million people are thought to participate regularly in ‘everyday creativity’ or non-formalised art activities ^2^. In addition, more formal arts programmes take place in a variety of settings, some with a specific health focus (often in conjunction with social prescribing schemes), and others without, although they may still impact health and wellbeing.

Access to and engagement in art is a fundamental human right, under Article 27 of the Universal Declaration of Human Rights (UNHR), and creativity can be seen as an intrinsic part of what it means to be human ^3^. Creative arts interventions have the potential to build social capital in communities, and to harness the positive power of community agency, improving public health ^4– 6^ Creative arts interventions may offer economic benefits for society and can reduce pressure on an overburdened health system ^2, 7^. At an individual level, the benefits of participating in creative arts are well-recognised and include lower mental distress, increased social connection, improved quality of life *,* personal growth and empowerment ^2, 7– 12^.

Against this background, there is an international policy drive calling for stronger pathways between arts, health and social care. This recognises the importance of realising the full potential for arts in public health policy and their role in helping achieve the UN Sustainable Development Goals ^13, 14^. Countries are shifting towards economic policies which incorporate wellbeing, recognising the impact of this on root causes of mental ill health, and creativity has been cited as a skill of the future by the World Economic Forum ^15^. ( https://www.weforum.org/agenda/2022/08/free-thinking-boosts-creativity/) In the UK, a recent All Party Parliamentary Group on Arts and Health has emphasized the value of arts to health, and called for a cross party departmental Creative Health Strategy ^2^.

### Wellbeing in older people

Like many populations worldwide, the UK population is ageing; currently 11 million people (19% of the population) are over 65 and this number is increasing ^16^. However, the number of years spent living in good health is falling ^2^. There are multiple reasons for this, but particular challenges facing older people may include declining social networks, loneliness, physical health challenges, loss of partners, caring roles, economic challenges and ageism ^17– 20^. Compared with previous generations, families are often more geographically distant than before, with implications for community networks and support ^21^. With regard to mental health, there is an unmet need for support: depression affects around 22% of men and 28% of women over 65, yet it is estimated that 85% of older people with depression receive no help at all from the NHS *( https://www.mentalhealth.org.uk/explore-mental-health/statistics/older-people-statistics)*.

Whilst mental health and wellbeing are interrelated concepts, they may also be discrete; one can be mentally ill whilst still achieving a state of subjective wellbeing; conversely one could lack a state of wellbeing without being mentally ill. Previous studies in this field have tended to focus on how creative arts interventions impact patient groups with specific diagnoses e.g. mental illness, dementia, cancer etc ^22^.This review will look at individuals living in the community who have not been included in arts interventions by virtue of a particular diagnosis (e.g. mental ill-health, dementia etc.), and examine impact of creative arts interventions on individual wellbeing.

We recognise that wellbeing is a difficult concept to define and will mean different things to different individuals. Following the literature in this field, we define wellbeing as a state of an individual ‘living well’, which may include elements of experienced, evaluative and social wellbeing ^23^. Wellbeing is an important aspect of health; the WHO defines health as ‘a state of complete physical, mental and social wellbeing and not merely the absence of disease or infirmity’ ^24^; This is a bold definition, and such a comprehensive state of wellbeing may be difficult to achieve, yet it remains an important goal; interventions which may facilitate movement towards an improved state of wellbeing are key to individual and community health.

### Towards a theory of creative arts and wellbeing

Despite the recognised health benefits of participating in creative arts, it remains unclear exactly how creative arts interventions impact participants’ wellbeing. There is a recognised need for better theoretical understanding of how elements of creative arts programmes impact wellbeing amongst older people, and in particular when, how, for whom and to what extent they work ^2, 18, 23, 25– 27^. With this in mind, our review seeks to answer the question *‘in what contexts and by which mechanisms can creative arts interventions impact wellbeing in older people?’.* We will use a realist approach, which is well-suited to complex interventions with multiple components, such as creative arts programmes. We will look at participatory arts interventions, defining these as an intervention where an individual has an opportunity to actively take part in the creative process e.g. by painting or writing or singing. We will also seek to uncover possible negative impacts of interventions, as well as focusing on subgroups such as men. The knowledge gained should be helpful to those planning and taking part in interventions, and will add to a growing theoretical understanding of this field.

## Aim

This project aims to synthesize the evidence on how elements of creative arts programmes improve wellbeing amongst older people, and in particular when, how, for whom and to what extent they work. Using this knowledge, we hope to develop guidance for providers who want to use creative arts interventions to improve the wellbeing of older people living in the community.

## Objectives

1. Conduct a realist review of the existing literature to develop an in-depth understanding (captured in a realist programme theory) of the use of the creative arts to improve the wellbeing of older people living in the community: (Work Package (WP)1, months 1-9)
2. Use the knowledge within the realist programme theory to develop guidance for service providers of creative arts who want to use their services to improve the wellbeing of older people living in the community (WP2, months 10-12)

This project will run from September 2024 - August 2025.

## Methods

### Patient and Public Involvement

Our stakeholder group (comprising older people with lived experience of creative arts interventions, policymakers, service providers and commissioners) will be formally involved at each stage of this review. We will discuss our emerging findings and sense-check our developing theory with this group. Dissemination will be guided by our stakeholder group and will target a range of audiences, building on links with policymakers, commissioners, providers, and the public, and designed reach key actors within the field of creative health e.g. the National Centre for Creative Health and the Creative Ageing Development Agency. A final stakeholder event focused on knowledge mobilisation will aid development of recommendations.

This review (PROSPERO registration no: CRD42024580770) will follow Pawson’s five iterative steps for realist reviews. Realist review is well-suited to research on creative arts interventions because such interventions are complex and multi-faceted ^28^. Context is key to determining the relative success or failure of any given intervention, and the mechanisms underlying how context relates to outcome are not always clear. A realist approach allows us to identify and understand some of these mechanisms, building on work done by others in the field ^25, 29^. The review will be guided by the RAMESES publication standards for realist synthesis ^30^.

For the purposes of this review, the outcome is wellbeing, as defined by the authors of the primary literature; this may be measured by subjective or evaluative means.

***Step 1: locating existing theories***

**Figure 1.**
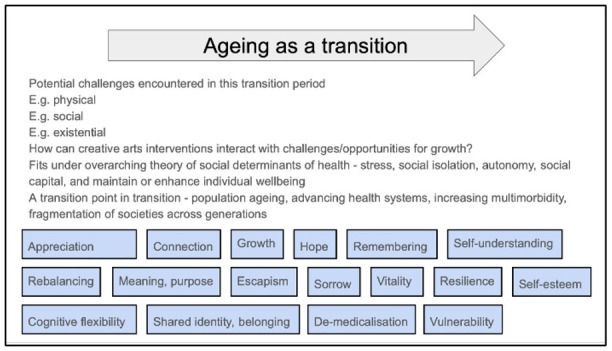
Sketch of initial programme theory. Overarching concept of ageing as a transition process at the top. Blue boxes identify concepts (possible contexts or mechanisms) which may be important in contributing to potential impact of creative arts interventions on wellbeing.

The first step of the review process will be to source existing theories within the field of creative health and from other disciplines which may shed light on the contexts and mechanisms at play in creative arts interventions for older people. Early discussions and literature scoping have informed an initial programme theory ( Figure 1), showing the possible contexts and mechanisms of interest.

***Step 2: searching for evidence***

The second step will involve searching the literature for relevant papers to include in the review. The search strategy will combine textword and subject heading terms for our key concepts of creative arts, wellbeing, older people and UK. Searches will identify literature published in the English language across ten databases (ASSIA (Proquest), CINAHL(EBSCOHost), Embase(OvisSP), Medline(OvisSP), PsycINFO(OvidSP, Science Citation Index, Social Science Citation Index, Arts & Humanities Citation Index, Conference Proceedings Citation Index - Science and Conference Proceedings Citation index - Social Science & Humanities (Web of Science Core Collection), without restriction based on study type or publication date.

The search strategy will be designed, piloted and conducted by an experienced librarian in collaboration with the rest of the project team. In addition to database searches, this will include citation tracking and searching grey literature, as it is likely that there are many creative arts programmes for older people which may not be formally evaluated within the scientific literature, but which would include valuable material for developing the programme theory. In line with the iterative nature of realist reviews, further searching will take place to provide more data for specific subsections of the programme theory as required.

***Screening***

References will be exported to Covidence where duplicates will be removed and screening will be undertaken first by title and abstract and full text by AC ( *Covidence. [Internet]. 2024 [20th August 2024]. Available from: https://www.covidence.org/*.). At both stages, a 10% random sample will be independently reviewed by another member of the research team, AF. Disagreements not resolved through discussion between the researchers will be resolved through majority vote within the research team. We will seek additional information from study authors where necessary to resolve questions about eligibility.

***Inclusion and exclusion criteria***

- Inclusion criteria
  • Intervention
    ◦ participatory creative arts programme run in a community setting in the UK
  • Population
    ◦ UK adults aged 65 and over
  • Outcome:
    ◦ wellbeing
- Exclusion criteria:
  • Intervention
    ◦ creative arts programmes with receptive involvement (where the individual has a receptive role in the activity e.g. going to a museum, watching a film)
    ◦ programmes run in non-community settings (e.g. nursing or care settings)
    ◦ programmes run in countries other than England, Wales, Scotland, Northern Ireland
    ◦ programmes involving activities such as gardening, cooking and volunteering (following a consensus in the literature that these are not generally considered as creative arts activities, though we recognise that this may be debatable) or with an intergenerational focus
  • Population
    ◦ older people selected for inclusion in programmes by virtue of a specific diagnosis (e.g. dementia, cancer, mental ill health) or carer role
    ◦ or previous profession (e.g. veterans, music therapists)
    ◦ older people living in a non-community setting (care home, nursing home, or inpatient in hospital etc.)
  • Outcome
    ◦ programmes looking at outcomes other than wellbeing

***Step 3: article selection***

Full text articles will be selected for inclusion in the review based on relevance and rigour. Relevance will be determined by what the article is able to offer towards the development of the programme theory. Rigour will be assessed in two ways - at the level of the quality of the methods used in included articles to generate relevant data and at the level of the explanatory value of the programme theory. We will undertake quality assessment of the methodological quality of included articles only when we find that a particular article has contributed a substantial amount of data to our CMOCs or programme theory, or when a CMOC draws heavily on one article. We will judge the explanatory quality of the programme theory using criteria of consilience, simplicity and analogy. Briefly, consilience refers to the ability of any explanation to account for as much of the data as possible. Simplicity is based on Occam’s Razor and thus expects the theoretical explanations to be as simple as possible with minimal to no ad hoc exceptions. Finally analogy refers to the whether what we have found fits in with existing knowledge ^31^. A random sample of 10% of the included articles will again be independently reviewed by a second member of the research team, AF. As previously, any disagreement not resolved by discussion will be decided upon by majority vote within the research team.

***Step 4: extracting and organising data***

Data will be extracted by AC. Descriptive data from the included studies will be inputted into an Excel spreadsheet. These descriptive data will include details of the study (authors, publication date, country), participants (number, age), intervention (type of activity, setting, duration, referral pathway, funding) and outcome (measurement, findings). Data extracted for analytic purposes to develop and test (confirm, refute or refine) the CMOCs and emerging programme theory will be inputted into NVivo ( *Lumivero (2023) NVivo (Version 14) www.lumivero.com*), after rereading the full texts of the papers. The papers will initially be thematically coded in NVivo, both inductively (to enable the development of new ideas for the programme theory), deductively (based on the concepts contained within the initial programme theory) and retroductively (to infer what may be functioning as mechanisms). Codes will be refined through discussion as above, and interpretations and judgements agreed upon with the rest of the team, and at key points with the stakeholder group. As refinements are made, included papers will be reassessed for further contribution to the developing theory.

***Step 5: synthesising the evidence and drawing conclusions***

The analysis will then proceed to develop realist causal explanations for outcomes that are relevant to the programme theory, within each theme. The analysis will use a realist logic of analysis, moving between the data and the theory to explore how creative arts impact wellbeing in older people. The analysis will use interpretations of the coded data to build causal explanations that take the form of Context (C), Mechanism (M) and Outcome (O) configurations (CMOCs). The final programme theory will contain CMOCs that explain in which contexts certain mechanisms are triggered to produce outcomes relating to wellbeing, and consider the relative importance of these contexts. Our stakeholder group will provide feedback on the final programme theory.

## Ethics and consent

As this realist review is a secondary evidence synthesis of existing literature, ethical approval was not sought; however, this research will be conducted in full compliance with the Declaration of Helsinki on medical research and the UK Policy Framework for Health and Social Care Research ^32^.

## Data Availability

**Figshare:** PRISMA-P checklist for ‘In what context and by which mechanisms can creative arts interventions improve wellbeing in older people? A realist review protocol’. https://doi.org/10.6084/m9.figshare.27302667.v1 ^33^. Data are available under the terms of the (CC0 1.0 Public domain dedication).
