# Supplementary material for: In what context and by which mechanisms can creative arts interventions improve wellbeing in older people? A realist review protocol
Source: NIHR Open Res. 2025 Jun 16;5:19. Originally published 2025 Mar 4. [Version 2] doi: 10.3310/nihropenres.13746.2 (PMC12368484; doi:10.3310/nihropenres.13746.2)
Supplement: Peer review history

# Reviewer report: Reviewer response for version 2

**DOI:** 10.3310/nihropenres.15242.r36026

**Published:** 2025-08-20

**Authors:** Julian West, Hannah Zeilig

**Affiliations:** 1 Royal Academy of Music, London, UK; 2 London College of Fashion, University of the Arts London, London, England, UK

As mentioned in our first review, this is a strong study protocol. We do feel that points 1 and 2 which were raised in our initial review have not been addressed and that the reasons for omitting the research on wellbeing, creative  arts and people with dementia could be clarified. 

 The more nuanced discussion of wellbeing is welcome.

 Points 4 and 5 have not been addressed.

 Point 6 has been addressed with the inclusion of practitioners in the stakeholder group.

 We understand that point 7 is difficult to address, but do feel the study would be strengthened by sense checking  the search strategy with the the wider stakeholder group.

 Finally point 8 is addressed in the introduction and in the description of the exclusion criteria.

Is the study design appropriate for the research question?

Yes

Is the rationale for, and objectives of, the study clearly described?

Yes

Are sufficient details of the methods provided to allow replication by others?

Yes

Are the datasets clearly presented in a useable and accessible format?

Not applicable

Reviewer Expertise:

NA

We confirm that we have read this submission and believe that we have an appropriate level of expertise to confirm that it is of an acceptable scientific standard.

# Reviewer report: Reviewer response for version 1

**DOI:** 10.3310/nihropenres.14930.r34945

**Published:** 2025-05-10

**Authors:** Vicky Karkou

**Affiliations:** 1 Edge Hill University, Ormskirk, UK

This is a very well written piece that provides a strong rationale and study design consistent with the approach adopted.  Some minor comments for improvement are:

 There is a good overview of arts-based interventions.  The text sets out the whole field but it is only later on (towards the end of the introduction) that it becomes clear that the review is concerned with participatory arts interventions only.

 It is now clear how the RAMESES quality standards will be used (and not used consistently within the text: are you referring to quality standards or publication standards?).

 It will be good practice to move the websites from the text and reference website as a normal reference following relevant conventions.  

 p.4. I would not refer to men as a subgroup, but maybe simply a group.

Is the study design appropriate for the research question?

Yes

Is the rationale for, and objectives of, the study clearly described?

Yes

Are sufficient details of the methods provided to allow replication by others?

Yes

Are the datasets clearly presented in a useable and accessible format?

Not applicable

Reviewer Expertise:

Arts-based interventions, systematic literature reviews, realist evaluations, mixed methods researcher.

I confirm that I have read this submission and believe that I have an appropriate level of expertise to confirm that it is of an acceptable scientific standard.

# Reviewer report: Reviewer response for version 1

**DOI:** 10.3310/nihropenres.14930.r35074

**Published:** 2025-04-23

**Authors:** Julian West, Hannah Zeilig

**Affiliations:** 1 Royal Academy of Music, London, UK; 2 London College of Fashion, University of the Arts London, London, England, UK

In this article, the authors present the argument that despite the growing interest in the potential for the creative arts to support wellbeing, understanding concerning the mechanisms whereby these interventions impact wellbeing for older adults is poorly understood.  They also describe additional reasons for why older people should have access to art. The use of a realist review is proposed, involving context-mechanism-outcome configurations (CMOCs) to create programme theories which can be used to support those who provide services aimed at improving the wellbeing of older people. The methods of the proposed study are described in detail and seem both appropriate for addressing the research question and rigorously designed. We agree that there are recognised health benefits to participating in the creative arts, but as outlined by Clift et al (2021, 2025) there is a need for more robust, systematic work in this area.

 We would like to offer a few observations which may further strengthen this study.

  

1. Whilst we agree that there is not a great deal known about how creative activities affect wellbeing, we are aware that this has been of increasing interest in the last decade. For instance, Windle, G., et al., 2018 (Ref 3) explore the theoretical foundations of visual art programmes for people with dementia using realist synthesis; Cousins, E, et al., 2020 (Ref 4) created a taxonomy of arts interventions again drawing on realist methodology; there is also multi-disciplinary research into the benefits of music for people with dementia - see for example the work of Särkämö. The assumption that research pertaining to people living with dementia might not be helpful in informing work for all older adults seems problematic.
2. Following on from the previous point, we would question and appreciate further clarity on why this review excludes arts interventions that focus on older people with specific diagnoses (e.g. mental health, dementia or cancer.)  Although we agree that previous work has tended to focus on older people with conditions such as those outlined, we think that the excellent work in this area could still be usefully informative for understanding arts interventions and their impact on wellbeing for all older adults. We think it might be important to build on and extrapolate from work that has already identified some of the CMOCs that explain in which contexts certain mechanisms operate to effect wellbeing in arts interventions.
3. As wellbeing is a central focus for this paper a more nuanced and rigorous definition might be helpful. The concept of flourishing has also been identified as key for wellbeing - especially in relation to the arts. For instance, how does wellbeing differ from quality of life and to what extent should loneliness and social isolation (the absence of these) also be considered? In addition, we are unclear what evaluative and social wellbeing refers to. Finally, the WHO definition is widely accepted but fails to acknowledge the ‘ill-being’ that can be encompassed as part of wellbeing (Zeilig H, et al., 2019 (Ref 6)).
4. A stated aim is to develop guidance for providers; it would perhaps be useful to have a little more detail on the form that this might take and how this might be differentiated for a range of providers.
5. There is no mention of the different needs and contexts of people who are from global majority communities and how this will be considered, and we are unclear why men are mentioned as a ‘subgroup’ (is this due to demographic exigencies?)
6. In identifying the stakeholder group, we wonder why artists are not explicitly mentioned. The invaluable perspective of artists who lead groups for older people in a wide variety of contexts (often over long periods of time) should be fully integrated and yet is routinely overlooked (West, J., et al., 2024 (Ref 5)). This review could be strengthened by their inclusion.
7. The search strategy which will be conducted by an experienced librarian and the project team could also be sense-checked by the wider stakeholder group, especially older people with lived experience of arts groups.
8. With regard to the exclusion criteria, we query whether going to a museum can be considered a receptive activity in the same way as watching a film might be. This (as the authors do acknowledge) is highly debatable especially since many museums include activities that encourage creative engagement. Overall, we wonder whether a creative arts activity can be clearly distinguished as being EITHER receptive OR participatory. These distinctions are complex and could be clarified.

References:

- Clift, S., et al, 2021 (Ref 1)
- Grebosz-Haring, K. et al., 2025 (Ref 2)
- Windle, G., et al., 2018 (Ref 3) 
- Cousins, E, et al., 2020 (Ref 4)
- West, J., et al., 2024 (Ref 5)
- Zeilig H, et al., 2019 (Ref 6)

Is the study design appropriate for the research question?

Yes

Is the rationale for, and objectives of, the study clearly described?

Yes

Are sufficient details of the methods provided to allow replication by others?

Yes

Are the datasets clearly presented in a useable and accessible format?

Not applicable

Reviewer Expertise:

NA

We confirm that we have read this submission and believe that we have an appropriate level of expertise to confirm that it is of an acceptable scientific standard, however we have significant reservations, as outlined above.

## References

[1] : The need for robust critique of research on social and health impacts of the arts. *Cultural Trends* .2021;30(5) : 10.1080/09548963.2021.1910492 442-459 10.1080/09548963.2021.1910492

[2] : The need for a critical perspective on arts and health research and evidence reviews.2025; 10.31235/osf.io/y7axd_v2 10.31235/osf.io/y7axd_v2

[3] : Exploring the theoretical foundations of visual art programmes for people living with dementia. *Dementia (London)* .2018;17(6) : 10.1177/1471301217726613 702-727 10.1177/1471301217726613 28914090 PMC6068961

[4] : A Taxonomy of Arts Interventions for People With Dementia. *Gerontologist* .2020;60(1) : 10.1093/geront/gnz024 124-134 10.1093/geront/gnz024 30840060

[5] : Making a living moment more resonant: an exploration of the role of the artist in co-creative work with people living with dementia. *Wellcome Open Research* .2024;8: 10.12688/wellcomeopenres.19357.3 10.12688/wellcomeopenres.19357.3 PMC1110953638779057

[6] : Co-creativity, well-being and agency: A case study analysis of a co-creative arts group for people with dementia. *J Aging Stud* .2019;49: 10.1016/j.jaging.2019.03.002 16-24 10.1016/j.jaging.2019.03.002 31229214 PMC6597952
